# Supplementary figures and images for: The Potential Mechanism of Bufadienolide-Like Chemicals on Breast Cancer via Bioinformatics Analysis
Source: Cancers (Basel). 2019 Jan 14;11(1):91. doi: 10.3390/cancers11010091 (PMC6357202; doi:10.3390/cancers11010091)

K

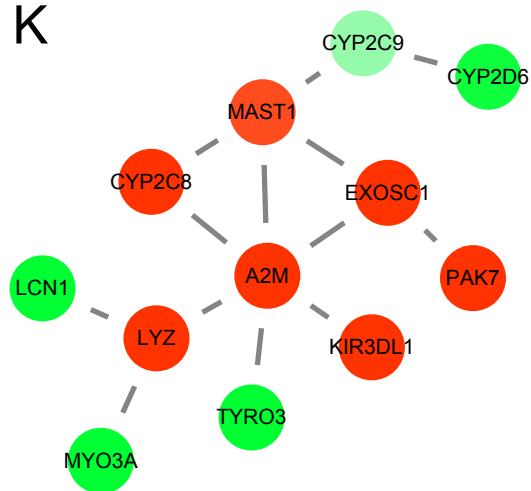

L

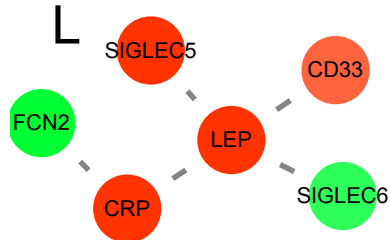

M

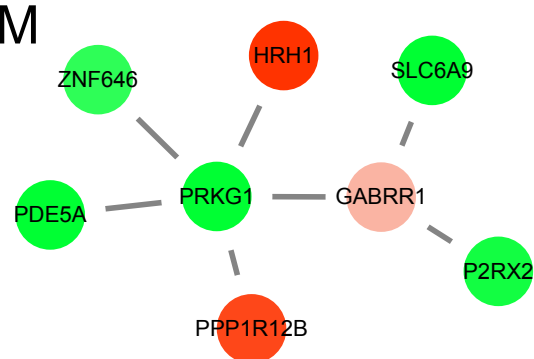

N

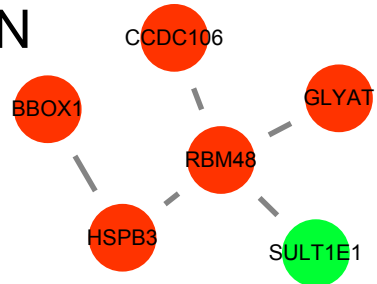

O

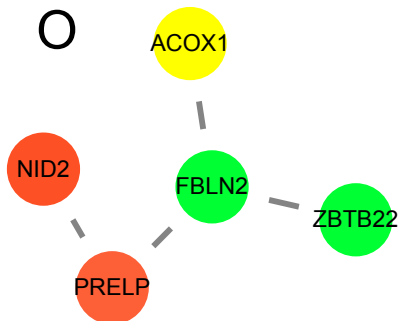

P

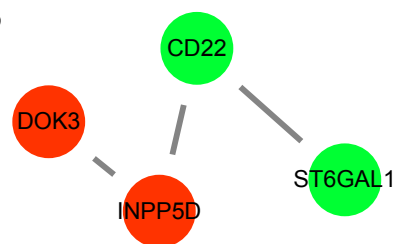

Q

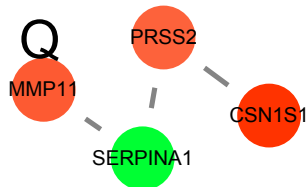

R

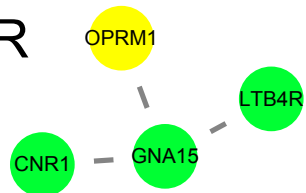

S

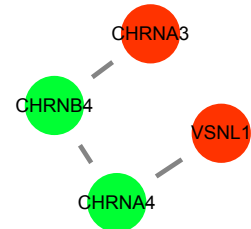

Supplement: Supplementary file 1 [file cancers-11-00091-s001.zip › Figure S1 Other differentially expressed networks regulated by bufadienolides-like chemicals.pdf]
